# Field and Laboratory Efficacy of Low-Impact Commercial Products in Preventing Olive Fruit Fly, *Bactrocera oleae*, Infestation

**DOI:** 10.3390/insects13020213

**Published:** 2022-02-21

**Authors:** Elissa Daher, Nicola Cinosi, Elena Chierici, Gabriele Rondoni, Franco Famiani, Eric Conti

**Affiliations:** Department of Agricultural, Food and Environmental Sciences, University of Perugia, 06121 Perugia, Italy; elissa_93d@live.co.uk (E.D.); nicola.cinosi@studenti.unipg.it (N.C.); elenachierici9@gmail.com (E.C.); franco.famiani@unipg.it (F.F.); eric.conti@unipg.it (E.C.)

**Keywords:** *Olea europaea*, olive fruit fly, Tephritidae, preventive methods, insect behaviour, deterrence

## Abstract

**Simple Summary:**

The adoption of sustainable methods for herbivore pest control has become mandatory in Europe, with the EU directive 128/09. Since then, stringent evaluation protocols have been applied to insecticides and several molecules (that are suspected to be unsafe for the environment or human health) have been banned. Hence, the evaluation of sustainable methods, e.g., preventive tools based on the manipulation of pest behaviour, must be considered. Using field and laboratory assays, we tested the efficacy of different products in preventing infestation of a key pest of olive orchards, the olive fruit fly *Bactrocera oleae*. Our findings may be useful for the development of control strategies in integrated pest management (IPM) and organic agriculture.

**Abstract:**

The olive fruit fly, *Bactrocera oleae*, is the key pest of olive trees in several areas of the world. Given the need for the development of sustainable control methods, preventive tools, based on the manipulation of pest behaviour, must be considered. Here, under field and laboratory conditions, we tested the efficacy of different products in preventing *B. oleae* infestation. A field trial was conducted, from July to November 2020, in an olive orchard located in Central Italy. A table olive variety was selected and sprayed with rock powder, propolis, the mixture of both, copper oxychloride, or water (control). All treatments, except propolis, caused a reduction of *B. oleae* oviposition in olives, compared to the control. The mixture allowed the strongest reduction of fly infestation throughout the season, suggesting a synergistic effect. Behavioural no-choice assays were conducted to better understand the effects of treatments on *B. oleae* females. Compared to the control, females showed a lower preference for the central area of an arena containing an olive twig bearing two olive fruits, fully developed, but still green, treated with rock powder, plus propolis mixture. For all treatments, *B. oleae* showed lower oviposition events, suggesting deterrence to oviposition. Our results indicate that the tested products may have value against *B. oleae*, within integrated pest management (IPM) and organic agriculture.

## 1. Introduction

Fruit flies, belonging to the family Tephritidae, include key pests of economically important fruit trees and vegetables [1]. Among the 932 species described to date [2], the most harmful ones are included in the genera *Bactrocera* Macquart, *Ceratitis* Macleay, and *Rhagoletis* Loew [3]. Economic damage is caused by larval feeding inside the fruits. Eggs are deposited under the fruit surface and larvae feed on the mesocarp causing fruit drop or a strong decline in fruit quality [4]. Many strategies have been deployed to manage these pests. Since the last four decades, organophosphate insecticides, especially as cover sprays, had been the most common control method adopted against fruit flies [5,6]. Organophosphate insecticides have a curative activity, mainly on eggs and larvae, as they can penetrate the outer layer of the fruit [7]. With the growing concerns about the impact of these insecticides on human health, environment, and non-target organisms, other pest management tools were promoted [8]. Since recent years, integrated pest management (IPM) strategies have been addressing the sustainable use of pesticides by improving monitoring systems in the agroecosystems [9]. The adoption of IPM or control methods authorized in organic farming have become mandatory in Europe (e.g., Directive 2009/128/EC on the sustainable use of pesticides). A regular pesticide review is periodically performed by the European Commission, and substances are banned if they are suspected to be unsafe for the environment or human health [9]. This is the case for malathion, applied extensively, with cover and bait sprays, to control *C. capitata*, and revoked with the Commission Implementing Regulation (EU), 2018/1495. Hence, alternative and safer substances for *C. capitata* control have been widely investigated (e.g., Commission Implementing Regulation (EU), 2019/1090) [10,11,12,13]. For many years, dimethoate represented the most effective product for controlling larvae of *Bactrocera oleae* (Rossi) (Diptera: Tephritidae) [14].

The olive fruit fly, *B. oleae*, is the key pest of cultivated olive tree, *Olea europaea* L. [15,16]. This species is almost globally widespread, ranging from Europe, Asia, and Africa to California and Mexico, where it is considered an invasive species [5,17,18]. Given the heavy use of chemicals, in the control of the olive fruit fly [19], alternative approaches are needed.

Biotechnical control strategies, such as mass trapping and attract and kill, have already been developed and are used to control low to medium infestation of *B. oleae* [5,6,7].

Kaolin particles and copper-based compounds can be used in organic and integrated pest management programs and may represent suitable control options. Kaolin provides an effective reduction of *B. oleae* oviposition in olive fruits [20]. Copper has a two-fold effect on *B. oleae*, i.e., adult repellence and/or antibacterial properties, which disrupts the symbiosis between larvae and bacterial symbionts [21,22,23]. Indeed, the endosymbiont *Candidatus* Erwinia dacicola (Enterobacteriaceae: Gammaproteobacteria) [24] is crucial for *B. oleae* larvae development in unripe olives [22]. This endosymbiont is involved in preventing the inhibitory effect of oleuropein [25], enabling olive fruit fly larvae to exploit the same host for an extensive period [22]. The use of copper in oliviculture is authorized for controlling plant pathogenic bacteria and fungi [26]. Therefore, the efficacy of copper against *B. oleae* should be considered as a side effect, albeit desired, of applications that target plant pathogens. Another compound, propolis, might be effective against *B. oleae*, due to its demonstrated antibacterial properties and, possibly, oviposition deterrence [21,27].

Thus, improving control protocols, using sustainable products, characterized by oviposition deterrence effects on *B. oleae*, becomes essential. Under field and laboratory conditions, we evaluated the efficacy of two new commercial formulations, based on propolis and rock powder, both allowed in organic agriculture and of traditional copper formulations. In the field, we assessed the efficacy of these products in preventing *B. oleae* infestation, whereas, in the laboratory, we investigated the supporting mechanism.

## 2. Materials and Methods

### 2.1. Field Experiment

The trial was conducted in an olive grove located in Spello (Umbria, Italy; geographic coordinates 42°59′26″ N 12°41′47″ E), from July till November 2020, on table olives belonging to the Ascolana Tenera variety. Three olive trees were selected, and, for each tree, five branches (bearing 24 to 37 drupes) were treated with the different products. Treatments consisted of: rock powder (commercial name “Polvere di Roccia”, CIFO Srl, San Giorgio di Piano, BO, Italy), at the dose of 6 kg/ha (600 g/hl concentration); propolis (commercial name “Propolis”, CIFO Srl), at the dose of 3 L/ha (300 mL/hl concentration); a mixture of propolis, plus rock powder, at the dose of 3 L/ha (300 mL/hl) + 6 kg/ha (600 g/hl), respectively; copper oxychloride (commercial name “Cupravit Blu 35 WG”, Bayer Cropscience S.r.l.), at the dose of 4 kg/ha (400 g/hl concentration); and water for the experimental control. All products, except rock powder, were diluted with water. The doses were determined according to the information reported in the labels of the commercial products. Rock powder was applied for water suspension. This product is <20 μm, obtained by mechanical grinding, sterilization, and micronization of a selection of volcanic rock, and contains chabasite (70%), phillipsite (2%), K-feldspar (5%), biotite (2%), pyroxene (3%), and volcanic glass (18%). Concerning propolis, this product is an extract of honeybee propolis (1.2% *w*/*w*) in linseed oil solution (complete information available at www.cifo.it, accessed on 2 February 2022).

About 100 mL of each treatment were sprayed in each replicate (on both adaxial and abaxial surfaces of the leaves), until drip, to assure a complete coverage. Applications were repeated four times during the season and scheduled based on time after previous application (maximum one month) and product washing out by rain (on 17 July, 11 August, 24 August, and 2 September). The first application was conducted, in correspondence with the initial capture of *B. oleae* in pheromone traps, when no olives were infested yet. Thirteen periodical surveys were conducted from 29 July to 26 November. For each survey, all olives belonging to the treatment and control branches were carefully inspected to reveal any presence of oviposition punctures [28].

### 2.2. Insect Rearing

*Bactrocera oleae* adults were obtained from the larvae in infested olive fruits, collected on late September 2020 from different olive varieties of Umbria region (central Italy). Fruits were placed on a plastic net (1 cm × 1 cm hole size), suspended inside a mesh cage (Kweekkooi 40 cm × 40 cm × 60 cm, Vermandel, Hulst, The Netherlands), and maintained under controlled environmental conditions (25 ± 1 °C, 60 ± 5% R.H., 16:8 h L:D photoperiod). Two layers of paper towels were placed at the bottom of the cage. Mature larvae that dropped from the olives hid under the paper layers and pupated. Pupae were collected three times per week and transferred to smaller mesh cages (Kweekkooi 30 cm × 30 cm × 30 cm, Vermandel, Hulst, The Netherlands), until the emergence of adult flies. These were provided with tap water-soaked cotton balls and pure honey on a piece of Parafilm^®^. Food was refreshed every 2 days. Adults were maintained together for mating for about two weeks, before being used for the bioassays [29].

### 2.3. Laboratory Behavioural Assays

Behavioural no-choice tests, using female *B. oleae*, were conducted to investigate possible deterrence mechanism of the different products that were tested in the field. Olive twigs, of the Frantoio variety, were collected from the field, stored at 4 °C, and used within 3 days. Healthy twigs, of approximatively 3 cm length, each bearing two olive fruits, fully developed, but still green and a leaf, were sprayed until drip, with 2 mL of each tested formulation. Treatments consisted in rock powder, propolis, a mixture of propolis and rock powder, copper oxychloride, and copper sulphate (Poltiglia Caffaro 20 DF NEW-Sumitomo Chemical Italia s.r.l., used at 7 mg/mL).

Equivalent concentrations to the field were employed. Water was applied as the control. A 20 mL atomiser bottle (Shenzhen Jiawang Trading Co., Ltd., Shenzhen, China) was used to apply treatments on the olive twigs. After spraying, the twigs were allowed to air dry (approximately for 30 min).

The experimental arena consisted of a Petri dish (15 cm diam × 1.5 cm), set upside-down on a filter paper (Filter-lab 1510, Filtros Anoia, S.A. Barcelona, Spain), covering a treated twig placed in the centre. A circle, of 5 cm diameter, containing the twig, was drawn using a fine-tip pencil and considered the central area of the arena. For the behavioural test, a single *B. oleae* mated female was introduced under the Petri dish. After two minutes of acclimation [30,31], each female was observed for 10 min, and its behaviour was recorded using JWatcher 1.0 [32,33]. Recorded behaviour included the entrance of the insect in the central area of the arena, exit from it, and oviposition events. If oviposition was observed, the fruit was dissected at the end of the bioassay and observed under a stereomicroscope to verify egg presence. Each twig and arena were used to test two females, unless a fruit was punctured, in which case the twig was replaced with a new one. The bioassay room was kept at an average temperature of 25 °C (±1). For each treatment, 92 females were tested.

### 2.4. Statistical Analysis

For field data, the effect of treatments on the cumulative number of *B. oleae* having infested fruits (calculated on % of total observed fruits) was evaluated, by means of two-parameter asymptotic models [34]. To account for repeated observations across space (multiple treatments conducted on the same plant) and time (repeated observations on the same branches), the plant identity effect was included as a random term in the model, and an autocorrelation structure between consecutive samplings was considered [35]. The number of olives per each treated branch was recorded at each observation. Sometimes, the number of olives at a given sampling date was lower, compared to the number of the previous survey. This was because infested fruits may drop during the season [5,36], and this phenomenon increases with *B. oleae* infestation [37,38]. We have occasionally observed dropped fruits at different timings. Since most of them exhibited *B. oleae* oviposition marks, we assumed, for data analysis, that all dropped fruits were infested by *B. oleae*. Differences between treatments were evaluated by means of the likelihood ratio test (LRT) [34,39].

For laboratory data, several common indexes were calculated, including: residence time (i.e., the time spent by each female in the central area of the arena), percentage of active insects (i.e., the percentage of insects that visited the central area of the arena), and time to visit, defined as the time period from the start of the observations until the entrance of *B. oleae* inside the central area [40]. Residence time data were subjected to arcsine square root transformation and analyzed by means of generalized linear model (GLM), with underlying Gaussian distribution. Treatment comparisons with the control were assessed by means of multiple comparisons procedures with Dunnett adjustment. Active insects were calculated by means of binomial GLM, followed by multiple comparisons procedure with Dunnett adjustment. Time to visits were evaluated using the Cox proportional hazard model (COXPH) [41,42]. The percentage of insect ovipositing in the treatments vs. control were analyzed by means of Fisher’s exact test (FET), due to the low number of positive outcomes in some treatments. The *p*-values were adjusted using Hochberg procedure. Data analysis was conducted using R [43], packages “nlme” [44], and “survival” [42].

## 3. Results

### 3.1. Field Experiment

**Figure 1:**
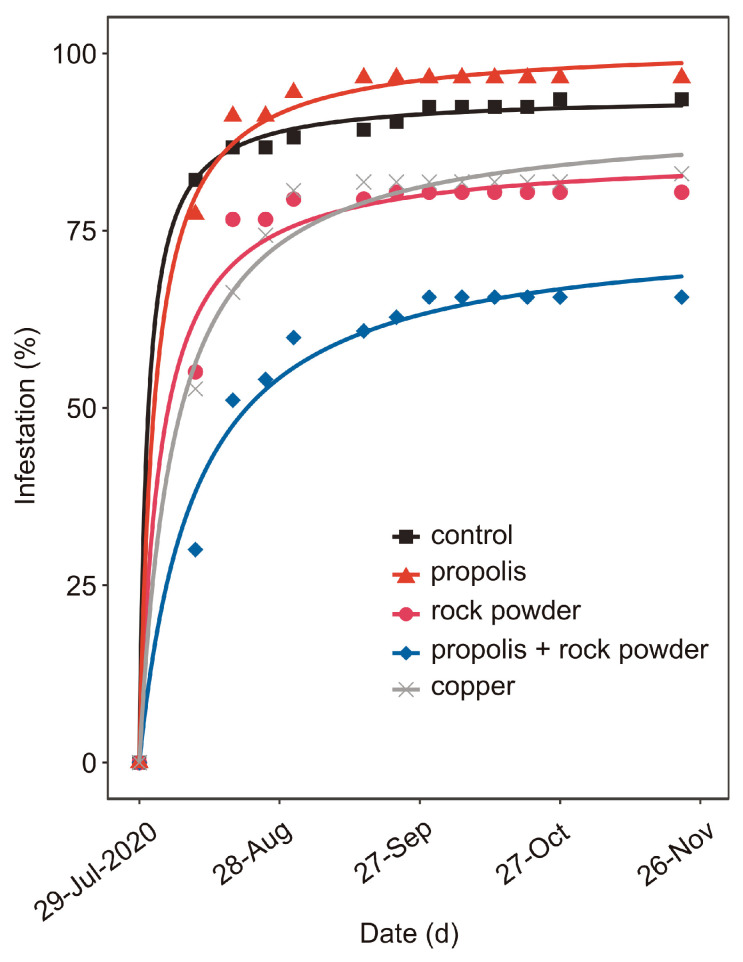
Field experiment. Seasonal trends of olive fruits, infested by *Bactrocera oleae*, on branches treated with rock powder (purplish red), propolis (red), a mixture of propolis + rock powder (blue), copper oxychloride (grey), or the control (black). Symbols represent observed mean % of raw data. Lines represent fitted two-parameter asymptotic models. According to likelihood ratio test, no differences at *p* ≤ 0.05 were detected between propolis and the control or between rock powder and copper.

The percentage of olives infested by *B. oleae*, i.e., showing at least one oviposition puncture, differed among experimental treatments (LRT, χ^2^_(8)_ = 63.28, *p* < 0.0001; Figure 1), with a very high level of observed infestations in control twigs twelve days after the first treatment (82.2% ± 10.55). Olive twigs, bearing two olive fruits, fully developed, but still green, treated with rock powder and copper oxychloride, exhibited similar levels of infestation (χ^2^ _(2)_ = 4.58, *p* = 0.10), and both treatments were effective in reducing *B. oleae* oviposition, compared to control (χ^2^ _(2)_ = 22.50, *p* < 0.0001) or propolis (χ^2^ _(2)_ = 12.17, *p* = 0.0023). The mixture of rock powder and propolis was also more effective, compared to propolis (χ^2^ _(2)_ = 38.02, *p* < 0.0001) or the control (χ^2^ _(2)_ = 49.37, *p* < 0.0001). Additionally, this mixture was more effective, compared to rock powder or propolis used alone (χ^2^ _(2)_ = 17.79, *p* < 0.0001). The effect of propolis did not differ from the control (χ^2^ _(2)_ = 3.26, *p* = 0.19).

### 3.2. Laboratory Behavioural Assays

**Figure 2:**
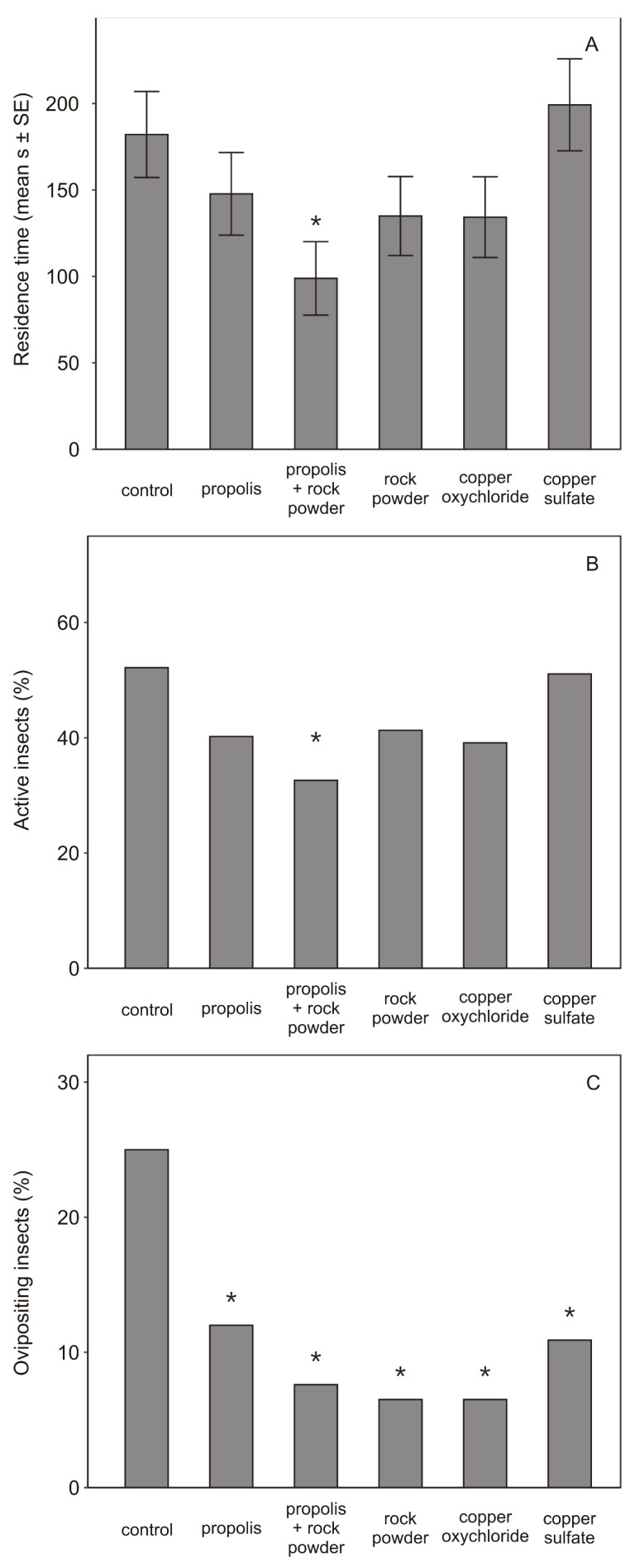
Behavioural assays. Residence time (mean s ± SE) (**A**) active insects (%) (**B**) and ovipositing insects (%) (**C**) of *Bactrocera oleae* in the central area of a Petri dish arena, containing an olive twig, bearing two olive fruits, fully developed, but still green, subjected to one of the following treatments: rock powder, propolis, a mixture of rock powder and propolis, copper oxychloride, copper sulfate. Water was used as the control. Differences between treatments and control were evaluated by means of generalized linear models (for residence time and active insects) or Fisher’s exact test (for ovipositing events), followed by multiple comparisons (asterisks denote statistically significant differences, *p* ≤ 0.05).

The residence time of the *B. oleae* females, in the internal area of the arena, varied depending on the treatment (Gaussian GLM: *F*_(5, 546)_ = 2.71, *p* = 0.020), with the mixture of rock powder plus propolis being lower than the control (*P*_Dunnett_ = 0.038; Figure 2). All other treatments were not significantly different from the control (*P*_Dunnett_ > 0.05). These results were also confirmed by data on active insects (i.e., the % of insects that entered the internal area), being lower for rock powder and propolis, compared to the control (binomial GLM, *P*_Dunnett_ = 0.032). *Bactrocera oleae* oviposition events were significantly lower for all treatments, compared to the control (FET, propolis: *P*_Dunnett_ = 0.036, rock powder and propolis: *P*_Dunnett_ = 0.007, rock powder: *P*_Dunnett_ = 0.004, copper oxychloride: *P*_Dunnett_ = 0.004, copper sulphate: *P*_Dunnett_ = 0.036).

Similarly, females showed a higher time to visit, i.e., they required more time before entering the central area of the arena, when olive twigs were treated with the mixture of rock powder and propolis, compared to the control (COXPH, *p* = 0.006, Table 1).

## 4. Discussion

The growing consciousness on the side effects of chemicals used for plant protection has resulted in increased research of more environment-friendly approaches, which encompass the manipulation (e.g., deterrence or repellence) of pest behaviour [45]. Such strategy has been investigated in several pest groups and can be either mediated by the plant through direct plant resistance or achieved using chemical compounds [46,47,48,49]. In our research, the oviposition deterrence efficacy of two new formulations was investigated, under field and laboratory conditions.

The field experiment results reported here demonstrate the role of the rock powder, especially when combined with propolis, in reducing fruit infestation by *B. oleae*. The cultivar Ascolana Tenera is known to be highly susceptible to fly infestation [50], due to its large size and greener colour, which make it more attractive, compared to other varieties [15,28]. This explains, at least partially, the high level of infestation on this variety at the beginning of the trial, compared to other varieties (data not shown). In spite of that, during the whole data collection period, i.e., from 12 days after first treatments to 11 weeks later, the branches treated with the rock powder and propolis mixture showed lower levels of infestation, compared to those of the other treatments. Particle film technology represents an important instrument in controlling insect pests, capable of interfering with insect behaviour and egg-laying [51,52]. This technology is also one of the few alternatives acceptable in organic olive groves for managing *B. oleae* [53]. A different type of rock powder, i.e., zeolite, showed a similar activity against several insect pest groups, including fruit flies (reviewed by [54]). The deterring effect is comparable to that of kaolin, a clay mineral widely used in preventing olive infestation by *B. oleae* [23,55,56,57]. Indeed, particle-film can act both on the host recognition ability of gravid *B. oleae* females and tactile modification of olive surface, potentially leading to oviposition deterrence [20]. On the other hand, with its antimicrobial action, propolis could potentially interfere with the strict relationship between bacteria and *B. oleae* [21]. The olive fruit fly exploits volatile and non-volatile molecules from epiphytic bacteria, in order to locate host plant and find bacteria, which are used as a food source by adult flies [58]. In spite of that, in our experiments, the treatments with propolis alone were not effective in reducing fly infestation, compared to the control. Therefore, we can hypothesize a synergistic effect of rock powder and propolis on *B. oleae* behaviour. In addition, based on observations in the field (data not shown), propolis could have served as an adjuvant, when mixed with rock powder, providing a longer persistence of rock powder on plants.

Treatment with copper oxychloride also resulted in a lower level of infestation, compared to control. This can be explained by the symbionticide and ovipositional deterrent actions of copper compounds [22,59]. However, copper accumulates in soil, the discovery of negative effects of copper-based compounds on soil microbiota, and the development of resistant strains have raised concerns about its use [60]. Therefore, national and international legislations have introduced maximum limits of copper use in agriculture, as well as alternatives for its gradual replacement, are under evaluation (e.g., Commission Implementing Regulation (EU), 2018/1981, [61]). In any case, negative effects on beneficial organisms must also be assessed for other compounds, such as clays. Kaolin-based products, similar to copper, can alter the behaviour, survival, or community composition of predators and parasitoids, such as Anthocoridae, Coccinellidae, and Braconidae [62,63]. These native and introduced entomophages often provide partial or substantial control of olive secondary pests, such as moths, scales, and psyllids [64,65]. Concerning soil biodiversity, a mixture of copper and propolis sensibly reduced the arthropod biodiversity in olive orchard soils, compared to the control [66]. However, in agroecosystems, such reductions can be more than counterbalanced by agricultural practices, such as cover crop mulching and no-tillage management, which have been demonstrated to support high invertebrate biodiversity (e.g., Collembola) [67,68,69]. In the present research, we have not evaluated the possible non-target effects on beneficial organisms of the high doses of the tested products (e.g., rock powder and copper), which remains unknown. Results of the behavioural assays are consistent with those of the field tests. The mixture of rock powder and propolis elicited, in *B. oleae*, the highest time to visit, lowest residence time, and lowest percentage of active individuals in the central area of the arena containing the treated twigs. This indicates a lower preference by the olive fruit fly, when both compounds are present in the environment, although additional investigations are necessary to define the behavioural mechanisms. Remarkably, all treatments reduced the percentage of oviposition events, by *B. oleae*, in olive fruits, compared to control, clearly indicating an oviposition deterrence effect of these products on the fly. These results confirm those obtained in the field experiments, except for propolis, which did not show any deterrent effect on the olive fruit fly in the field. This could be due to the high susceptibility of the variety used in the field, Ascolana Tenera, to *B. oleae* [50]. Additionally, it is worth mentioning that the oviposition events under laboratory conditions were in general low, which might have increased the deterrence efficacy of treatments. The low oviposition events might depend on the tested females, which were collected, reared, and assessed late in the season. Additionally, the laboratory rearing conditions and possible absence of bacteria, normally present in the olive orchards, might have decreased female fitness [21,70,71].

In conclusion, in both the field and laboratory, we demonstrated the positive effect of different products as oviposition deterrents against *B. oleae*, with the highest efficacy obtained from a mixture of rock powder and propolis. The efficacy seems to be amplified by a possible synergistic effect, although the actual mechanism needs further research and technical details, concerning their field application, are still under investigation. Since both rock powder and propolis are of natural origin and allowed in organic agriculture (CE 834/2007, 1235/2008), our findings encourage further large-scale field experimentation and implementation, with the aim of limiting the use of insecticides and copper-based products.

## Figures and Tables

**Table 1:** Behavioural assays. Summary of Cox proportional hazard model for time to visits of *Bactrocera oleae* in the central area of a Petri dish arena, containing an olive twig with fruits and leaves. Regression coefficient (β), standard error (SE), relative risk (exp(β)), and significance for the different experimental treatments (factorial levels of the covariate “treatment”), compared to the control, are given. A negative β value indicates the relative effect of the treatment, compared to control, on decreasing *B. oleae* visit probability in the internal area of the arena.

Covariate	(Level)	β	SE	Relative Risk of Visit Probability, Exp(β)	Z	*p*-Value
Treatment	(propolis)	−0.36	0.220	0.70	−1.62	0.105
	(rock powder and propolis)	−0.65	0.236	0.52	−2.763	0.006
	(rock powder)	−0.37	0.220	0.69	−1.691	0.091
	(copper oxychloride)	−0.39	0.222	0.68	−1.765	0.078
	(copper sulphate)	−0.03	0.207	0.97	−0.14	0.888

## Data Availability

The data presented in this study are available on request from the corresponding author.
